# A reinforced merging methodology for mapping unique peptide motifs in members of protein families

**DOI:** 10.1186/1471-2105-7-38

**Published:** 2006-01-25

**Authors:** Hao-Teng Chang, Tun-Wen Pai, Tan-chi Fan, Bo-Han Su, Pei-Chih Wu, Chuan-Yi Tang, Chun-Tien Chang, Shi-Hwei Liu, Margaret Dah-Tsyr Chang

**Affiliations:** 1 Institute of Molecular and Cellular Biology & Department of Life Science, National Tsing Hua University, Hsinchu, 30013, ROC, Taiwan; 2 Department of Computer Science, National Taiwan Ocean University, Keelung, 20224, ROC, Taiwan; 3 Department of Computer Science, National Tsing Hua University, Hsinchu, 30013, ROC, Taiwan

## Abstract

**Background:**

Members of a protein family often have highly conserved sequences; most of these sequences carry identical biological functions and possess similar three-dimensional (3-D) structures. However, enzymes with high sequence identity may acquire differential functions other than the common catalytic ability. It is probable that each of their variable regions consists of a unique peptide motif (UPM), which selectively interacts with other cellular proteins, rendering additional biological activities. The ability to identify and localize such UPMs is paramount in recognizing the characteristic role of each member of a protein family.

**Results:**

We have developed a reinforced merging algorithm (RMA) with which non-gapped UPMs were identified in a variety of query protein sequences including members of human ribonuclease A (RNaseA), epidermal growth factor receptor (EGFR), matrix metalloproteinase (MMP), and Sma-and-Mad related protein families (Smad). The UPMs generally occupy specific positions in the resolved 3-D structures, especially the loop regions on the structural surfaces. These motifs coincide with the recognition sites for antibodies, as the epitopes of four monoclonal antibodies and two polyclonal antibodies were shown to overlap with the UPMs. Most of the UPMs were found to correlate well with the potential antigenic regions predicted by PROTEAN. Furthermore, an accuracy of 70% can be achieved in terms of mapping a UPM to an epitope.

**Conclusion:**

Our study provides a bioinformatic approach for searching and predicting potential epitopes and interacting motifs that distinguish different members of a protein family.

## Background

Multiple protein sequence comparison can provide a valuable protein signature and, thus, contribute to the fields of structural biology and molecular evolution [1,2]. In general, sequence similarity identified by multiple sequence alignment (MSA) among a set of query sequences suggests similar function among the proteins [3]. These signatures can be readily obtained by web-based tools, such as BLAST [4], CLUSTALW [5], or MUSCA [6] systems. However, although the default parameters in most programs give satisfactory results, in some cases special variables need to be taken into consideration. For example, the allocation of major variations among a few query sequences can be achieved from the results of direct MSA, whereas the uniqueness of each sequence that is not well-aligned is difficult to reveal. It is also quite expensive and time-consuming to experimentally search for such unique peptide motifs (UPMs) that may involve the key biological functions of interest. Therefore, the need for effective and efficient identification of the UPMs located in a number of query proteins by novel bioinformatic tools is urgent. We have thus developed a reinforced merging algorithm (RMA) to rapidly identify the non-gapped UPMs for each member of a protein family.

In our study, the highly conserved human ribonuclease A (RNaseA) superfamily was initially tested to define the default input parameters. The RNaseA superfamily is composed of eight RNases including RNase1, 2, 3, 4, 5, 6, 7, and 8 with similar sequences and biological functions [7]. A pairwise comparison between RNaseA sequences shows more than 29% similarity; especially for RNase2 v.s. RNase3 and RNase7 v.s. RNase8, which have as high as 69.6% and 78.2% identities, respectively. Molecular evolutionary analysis revealed that both gene pairs emerged as a result of a relatively recent gene duplication event [7]. In addition to the RNaseA superfamily, other protein families with larger molecular weights including the members of epidermal growth factor receptor (EGFR), matrix metalloproteinase (MMP), and Sma-and-Mad related protein (Smad) family with respectively an average molecular weight of 150 kD, 50 kD and 55 kD were analyzed.

A monoclonal antibody (mAb) is commonly used in biological experiments for distinguishing the sequence specificity, quantity variation, and cellular localization of each member of the human protein families [8]. Generally, the epitope is composed of a charged and hydrophilic peptide of 7 to 20 amino acids exposed at the structural surface of the protein [9,10]. In this study, we generated one mAb specifically against RNase3 and the mapping experiments revealed that the epitope was indeed located at a unique region in RNase3 identified by RMA. Further analysis of several mAbs and polyclonal Abs against human RNaseA, EGFR, and MMP families demonstrated that they all recognized specific epitopes corresponding to the UPMs identified by RMA. We also mapped the UPMs on the resolved 3-D structures of the proteins and found that most of the UPMs were located on loop regions and exposed to the structural surfaces. Furthermore, the analytic comparison between RMA and PROTEAN [11], a commercially available software for prediction of antigenic regions, revealed that most of the UPMs identified by RMA matched well with the antigenic regions predicted by PROTEAN.

## Results

### Identification of the UPMs in the human RNaseA superfamily by RMA

**Figure 1:**
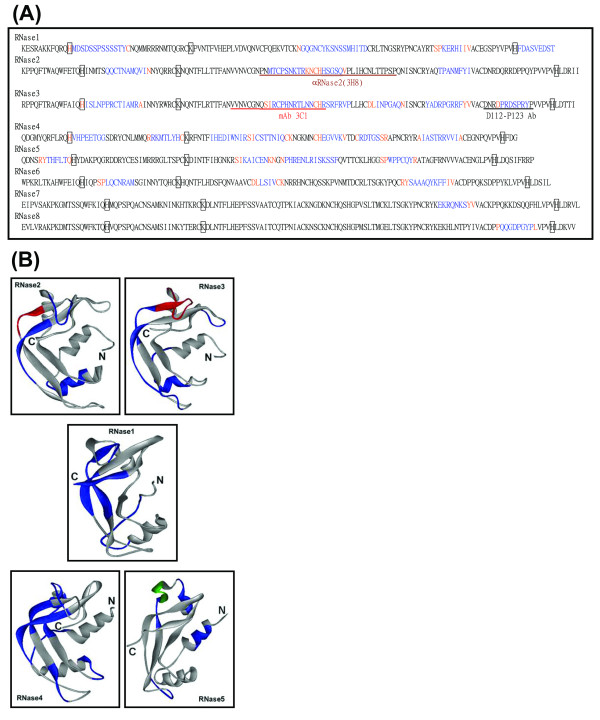
**The identification of UPMs among eight members of the human RNaseA superfamily by RMA**. (A) UPMs identified by RMA are marked as blue characters. The conservative residues at the ends of a UPM identified in the trimming phase are labeled in orange. Three key catalytic residues His, Lys and His responsible for the ribonucleolytic activity are displayed as open boxes in the conservative regions and shown in black characters. The brown, red, and black underline indicates the recognition sites for αRNase2, mAb 3C1 and D112-P123 Ab, respectively. The double underline represents the strict epitopes for αRNase2 or mAb 3C1. (B) The UPMs identified by RMA are labeled in blue on the crystal structures of mature human RNase1 (PDB: 1E21, 128 residues), RNase2 (PDB: 1GQV, 134 residues), RNase3 (PDB: 1DYT, 133 residues), RNase4 (PDB: 1RNF, 119 residues), and RNase5 (PDB: 1B1I, 127 residues). The epitopes of αRNase2 (H73-Q77) and mAb 3C1 (Q58-R73) are labeled in red. The identified UPM involved in the protein-cell interaction and angiogenesis in RNase5 is labeled in green (P64-F76).

The eight sequences of mature human RNaseAs were entered to RMA for analysis. As shown in Figure 1A, thirty UPMs of four to sixteen amino acid residues are identified and presented in blue. The orange characters at both ends of a UPM represent the trimmed residues in the trimming operators. The ones located between two blue sequence stretches reveal that the adjacent UPMs are not sequential.

Only one UPM could be identified in each of the most conservative RNase7 and RNase8, which is consistent with the phylogenetic analysis results of the human RNaseA superfamily, strongly suggesting a close relationship between RNase7 and RNase8 [7]. In addition, four and six UPMs were respectively found in RNase2 and RNase3, another highly conservative RNase pair. Likewise, in RNase1, RNase5 and RNase6, three to four UPMs could be located. Interestingly, as many as seven short UPMs were identified in RNase4. Twenty-five UPMs located on the 3-D structures of RNase1, 2, 3, 4 and 5 were labeled in blue (Figure 1B), and the loop coverage of each UPM was calculated (Table 1). It was found that fifteen UPMs were located at loop regions in the 3-D structures of RNase1, 2, 3, 4, and 5 with loop coverage greater than 50%.

It should be noticed that the key enzymatic active site residues correspondent to His12, Lys41 and His119 of mature RNase1 have been found to be located in the well-conserved regions among all eight RNases (Figure 1A, open boxes), indicating that RMA is practical in distinguishing the UPMs from the conserved sequences in a protein family. The UPM P116-Y122 located in the last loop of the *C*-terminus of RNase3 has been determined as the epitope for a polyclonal Ab, D112-P123 Ab, which specifically recognized RNase3 and was generated by Boix [12]. The UPM P64-F76 in RNase5 was located within a sheet-turn-sheet conformation involved in the protein-cell interaction (Figure 1B, labeled in green) [13].

### Application of RMA on protein families with larger molecular weight

**Figure 2:**
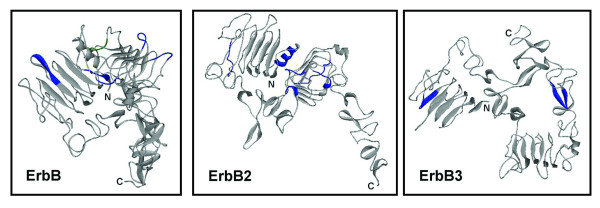
**Localization of the identified UPMs in ErbB, ErbB2 and ErB3**. The identified UPMs are labeled in blue on the crystal structures of extracellular domains in ErbB (PDB: 1NQL, 1210 residues), ErbB2 (PDB: 1N8Z, 1255 residues) and ErbB3 (PDB: 1M6B, 1342 residues). The EGF binding site on ErbB is labeled in green (S350-I356).

We have further examined the application of RMA on more protein families. The complete protein sequences of four members of human EGFR family ErbB, ErbB2, ErbB3 and ErbB4 were analyzed by RMA, and forty UPMs were identified. Fourteen UPMs located within the resolved 3-D structures at the *N*-terminal extracellular domains of approximately 530 residues in ErbB, ErbB2 and ErbB3 were labeled in blue (Figure 2). In ErbB, ErbB2 and ErbB3, six, six and two UPMs of six to thirteen residues in length were identified, respectively. Nine UPMs were found to possess loop coverage higher than 50% (Table 1). A UPM S350-I356 in domain III (L2) of the extracellular domain in ErbB was also identified as an EGF binding site for transduction of the ErbB signaling [14]. Another UPM in ErbB2, H42-Q51, matched nicely within the epitope of the anti-ErbB2 *N*-term polyclonal Ab (P36-Q51), an antibody that specifically distinguishes ErbB2 from the other members of the EGFR family (Stratagene Corp., Cat: B50175).

The sequences of mature human MMP1, 3, 8, 10, 12 and 13, which are categorized as members of the same subfamily and contain approximately 470 amino acids, were analyzed by RMA and forty five UPMs were identified. The results shown in Table 1 revealed that nine and six UPMs of five to ten residues in length were identified in MMP1 and MMP3, respectively. Currently only the structure of one of the catalytic domains (F1-P168) of MMP3 was determined in which two UPMs, Q103-T111 and T145-R151, could be identified by RMA. The former matched nicely with loop 7 and the latter matched well with loop 8 in the MMP3 structure [15]. The UPM R273-I278 of MMP1 overlapped with the epitope for a mAb αMMP1 (3B6) (residues S267-H277) (Santa Cruz, Cat: sc-21731). Likewise, a UPM K218-P225 of MMP3 matched perfectly with the characterized epitope for another mAb αMMP3 (1B4) (residues K218-H228) (Santa Cruz, Cat: sc-21732). In addition, the enzymatic active sites of MMPs in the highly conserved motif DDXXGIXXXYG (residues D152-G162) [16] and Gln120 [17] numbered correspondent to MMP1 were characterized to be located within the conserved regions, consistent with the results for the human RNaseA superfamily (data not shown).

Eight human Smads were analyzed by RMA and twenty four UPMs were identified. Currently only the *C*-terminal structures of approximately 230 residues of Smad2, Smad3 and Smad4 are available. Since the identified UPMs in Smad1 and Smad2 were located in the regions with unresolved structures, only the UPMs identified in Smad4 were shown in Table 1. The UPM G510-I518 was completely located in the L3 loop responsible for the binding with Ski protein, an oncoprotein that represses the TGF-β signaling. Another UPM E538-L551 with 64% of loop coverage in the last loop within the MH2 domain in the *C*-terminus of Smad4 was responsible for the interaction with the TGF-β receptors [18]. In the Smad superfamily, Smad4 plays a central role as it is the shared hetero-oligomerization partner of other Smads and it possesses the DNA binding ability for regulation of gene expression [19]. Within the DNA binding domain of Smad4, K45-K110, only one UPM T62-H67 was identified by RMA [20].

DOKs are phospho-proteins in the downstream of receptor tyrosine kinases. Six members of human DOK family, DOK1, 2, 3, 4, 5 and 6, of approximately 400 amino acids were analyzed by RMA and forty four UPMs were identified. A UPM Y402-G410 located at the *C*-terminus was found to be residing within the recognition site of an antibody αDOK2 (residues A393-K412) (Calbiochem, Cat: 506138); hence it is expected that αDOK2 can be used to distinguish DOK2 from the other DOKs (data not shown). Another protein family containing human carboxypeptidase D (CPD), E (CPE), M (CPM), N (CPN) was also analyzed. One of the UPMs in CPE matched well with the epitope for an anti-CPE mAb (BD Bioscience, Cat: 610758, data not shown). The analyses of EGFR, MMP, Smad, DOK, and CP families demonstrated that for members of protein families with large molecular weights, RMA still performed quite efficiently to provide useful structural and functional information.

### Application of RMA on homologous proteins from different species

The sequence comparison of homologous proteins among different species provides information on molecular and functional evolution. In this study, hRNase1 and its bovine homologue bpRNaseA were analyzed by RMA. The result shown in Table 2 revealed that six UPMs of six to eight residues in length were identified in each sequence. Ten out of the twelve identified UPMs were included in the loops, indicating that the loop regions in these two RNases were quite distinguishable. In addition, a UPM K31-D38 in bpRNaseA was included in the recognition site (S32-V43) of a polyclonal antibody α RNaseA generated by Younus and colleagues [21].

MsbA is a 630-amino acid prokaryotic homologue of human multidrug resistant protein. Five prokaryotic MsbAs from *E. coli*, *V. cholera*, *G. violaceus*, *S. oneidensis*, and *B. japonicum *were analyzed by RMA and fifty two UPMs were identified. Among which thirteen UPMs of five to seven residues identified by RMA in *E. coli *and *V. cholera *MsbAs were shown in Table 2. According to the 3-D structures of MsbAs of *E. coli *[22] and *V. cholera *[23], five of the identified UPMs were completely located in the loop regions and 67%–83% residues of two UPMs were covered by the loops. As a result, RMA has been demonstrated to be useful in analysis of homologous proteins among several species to rapidly identify the unique signatures of each query sequence.

### Specificity of mAbs against human RNase2 and RNase3

**Figure 3:**
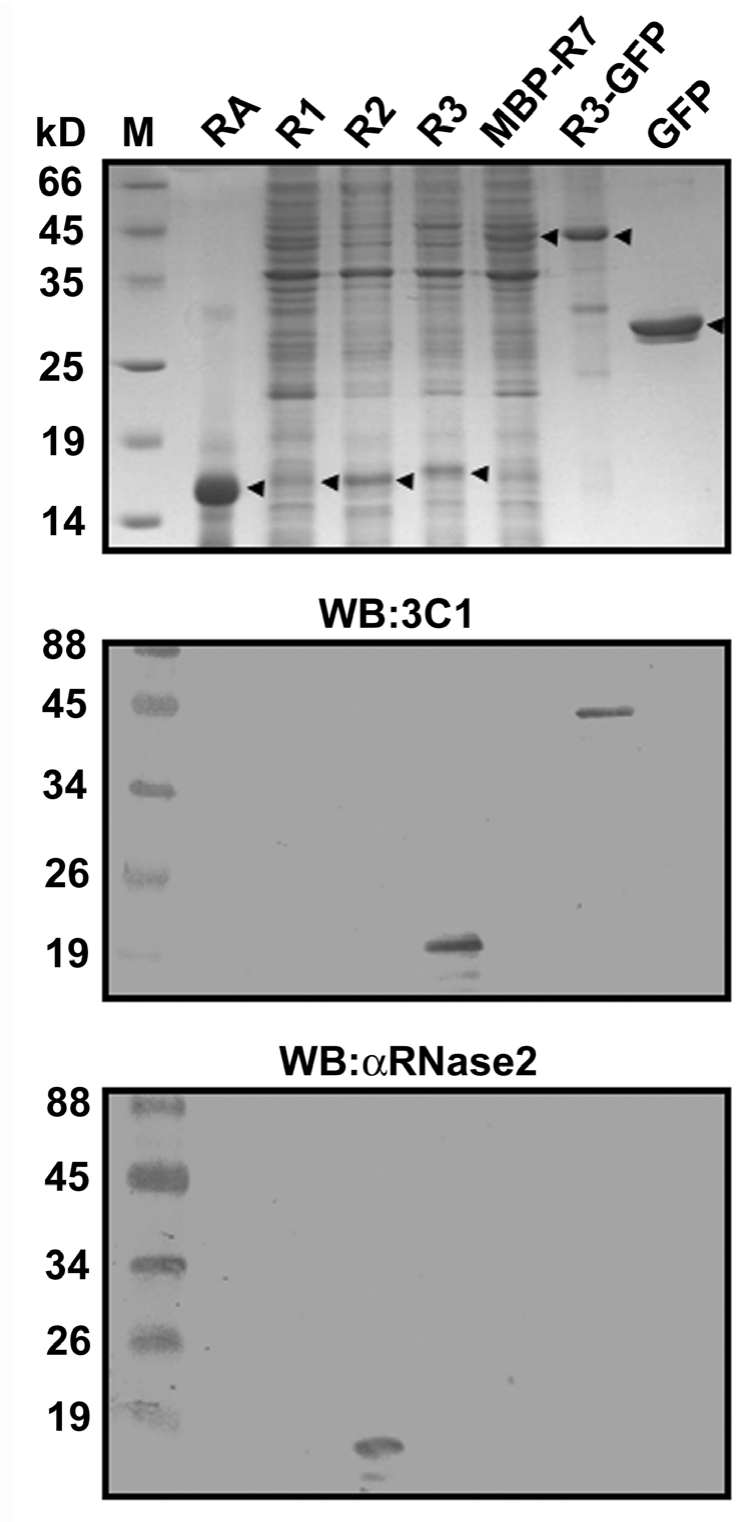
**Specificities of αRNase2 and mAb 3C1**. Bovine pancreatic RNaseA (RA), and bacterial lysates containing human RNase1 (R1), RNase2 (R2), RNase3 (R3), MBP-RNase7 (MBP-R7), RNase3-GFP (R3-GFP) and GFP were separated by 15% SDS/PAGE and analyzed by Coomassie Blue staining (top panel) as well as Western blotting using mAb 3C1 (middle panel) and αRNase2 (bottom panel).

One mAb against RNase3 named as 3C1 was successfully generated and purified. The specificity of mAb 3C1 and αRNase2, a commercially available mAb against RNase2, was further investigated. Bovine RNaseA (USB) and bacterial extracts containing recombinant human RNase1 (R1), RNase2 (R2), RNase3 (R3), MBP-RNase7 (MBP-R7), RNase3-GFP (R3-GFP), and GFP were separated by 15% SDS/PAGE and stained by Coomassie Brilliant Blue R-250 (Figure 3, top panel). The proteins were transferred onto a PVDF membrane and probed separately by mAb 3C1 and αRNase2 (Figure 3, middle and bottom panels). Our results demonstrated that mAb 3C1 could specifically recognize RNase3 and RNase3-GFP but did not cross-react with any other RNases or the expression tags (Figure 3, middle panel). Similarly, the αRNase2 only probed the recombinant RNase2 rather than the other RNases (Figure 3, bottom panel).

### Epitope screening for mAb 3C1 and αRNase2

**Figure 4:**
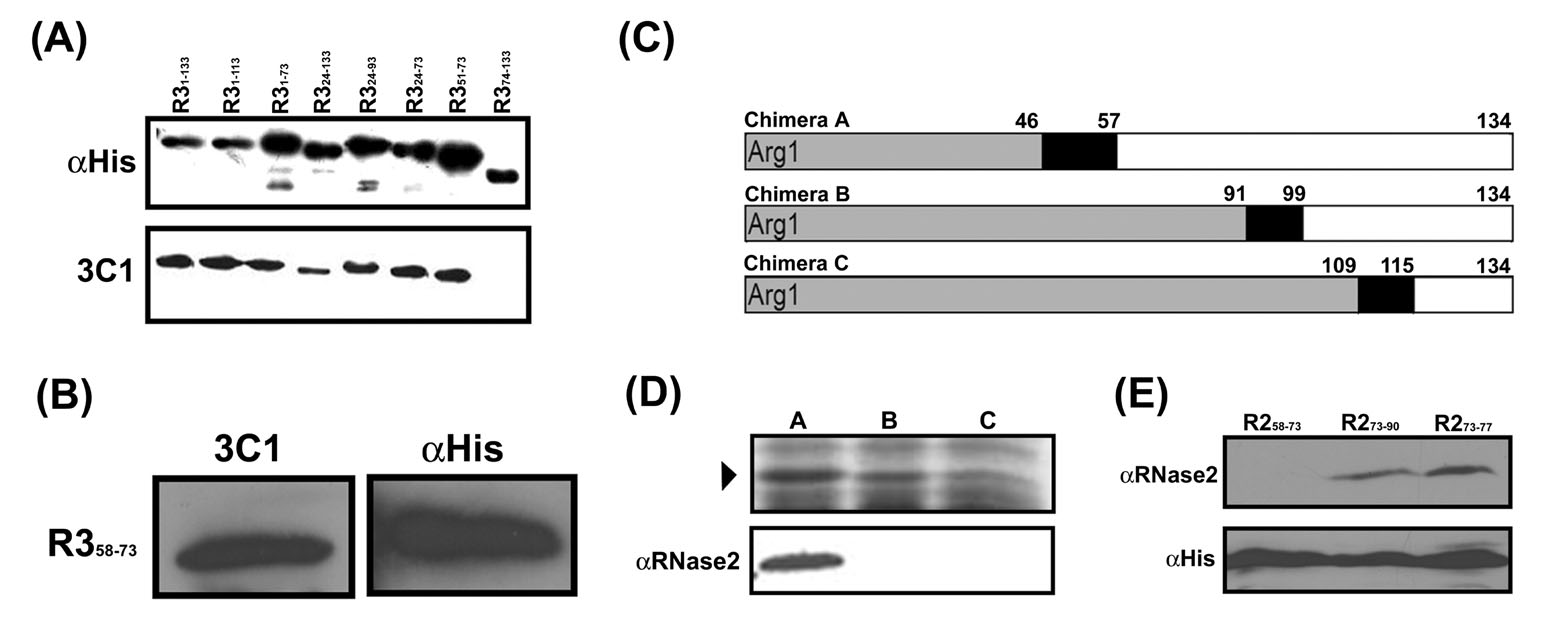
**Epitope screening for mAb 3C1 and αRNase2**. (A) The bacterial lysates containing RNase3-GFP (R3_1–133_), RNase3_1–113_-GFP (R3_1–113_), RNase3_1–73_-GFP (R3_1–73_), RNase3_24–133_-GFP (R3_24–133_), RNase3_24–93_-GFP (R3_24–93_), RNase3_24–73_-GFP (R3_24–73_), RNase3_51–73_-GFP (R3_51–73_), RNase3_74–133_-GFP (R3_74–133_) and (B) RNase3_58–73_-GFP (R3_58–73_) were separated by 12% SDS/PAGE and separately probed by αHis and mAb 3C1. A fragment linking R3_1–133_, R3_1–73_, R3_24–93_, R3_24–73_, and R3_51–73 _and GFP is 8.9 kD. (C) The schematic diagram of the chimeric constructions of RNase3/RNase2. The boxes labeled in grey, black and white indicate part of the $N$-terminus of RNase3, identical chimeric junctions and *C*-terminus of RNase2, respectively. Arg1 represents the first Arg residue in mature RNase3. (D) These chimeras were separated by 15{\%} SDS/PAGE (▶) and probed by αRNase2. (E) The bacterial lysates containing recombinant RNase2_58–73_-GFP (R2_58–73_, RNase2_73–90_-GFP (R2_73–90_) and RNase2_73–77_-GFP (R2_73–77_) were expressed and analyzed by Western blotting using $\alpha$ RNase2 and αHis.

To further identify the epitopes residing on RNase3 and correlate the experimental data with the identification by RMA, the mature and several truncated RNase3 were fused to GFP tags to efficiently express recombinant proteins including RNase3-GFP (R3_1–133_), RNase3_1–113_-GFP (R3_1–113_), RNase3_1–73_-GFP (R3_1–73_), RNase3_24–133_-GFP (R3_24–133_), RNase3_24–93_-GFP (R3_24–93_), RNase3_24–73_-GFP (R3_24–73_), RNase3_51–73_-GFP (R3_51–73_), and RNase3_74–133_-GFP (R3_74–133_). Upon induction with IPTG, these proteins were expressed and recognized by αHis as a positive control (Figure 4A, top panel). After immunoblotting with mAb 3C1, all recombinant proteins except the last one were specifically recognized, suggesting that the epitope for mAb 3C1 was located in the overlapping region within RNase3_51–73 _(Figure 4A, bottom panel; Figure 1A, red underline), a region that is possibly the most distinguishable between RNase2 and RNase3.

According to the identification by RMA, residues 51 to 57 of RNase3 were identical to those of RNase2; hence this region was further deleted to generate a recombinant RNase3_58–73_-GFP (R3_58–73_) protein. As expected, Figure 4B clearly showed that such a recombinant protein was probed by both αHis and mAb 3C1, indicating that the epitope for mAb 3C1 was indeed located within RNase3_58–73_, consistent with the identification by RMA (Figure 1A, red double underlines).

The epitope for αRNase2 was not indicated in either its manufacture or other reports. To allocate the epitope region experimentally, three clones containing chimeric RNase3/RNase2 were generated to express the chimeric proteins in *E. coli *(Figure 4C). The chimeric junctions contain three separate regions where the residues are identical between RNase2 and RNase3. Immunoblotting analysis revealed that only chimera A was recognized by αRNase2, indicating that P58–P90 of RNase2 was quite important in determining the antigenicity (Figure 4D; Figure 1A, brown underline). Based on the RMA and experimental results, two recombinant clones RNase2_58–73_-GFP (R2_58–73_) and RNase2_73–90_-GFP (R2_73–90_) were further generated. The results showed that the former was not probed by αRNase2, whereas the latter was specifically recognized (Figure 4E, lanes 1 and 2). Since only five continuous residues in RNase2_73–90_-GFP were predicted to be unique by RMA, the recombinant RNase2_73–77_-GFP (R2_73–77_) protein was further expressed and tested. Our result demonstrated evidently that a segment as short as only five residues of RNase2 were detected by αRNase2, indicating that the epitope for αRNase2 was successfully narrowed down to "H_73_SGSQ_77_" (Figure 4E, lane 3; Figure 1A, brown double underlines).

### Comparison of RMA and PROTEAN

**Figure 5:**
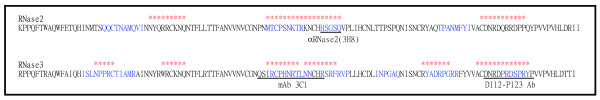
**Analyses of RNase2 and RNase3 by RMA and PROTEAN**. The RNase2 and RNase3 analyzed by RMA are extracted and modified from Figure 1A, and the UPMs are labeled in blue. The potential antigenic regions in RNase2 and RNase3 predicted by PROTEAN are labeled in red stars on top of each sequence. The epitopes of αRNase2, mAb 3C1 and D112-P123 Ab are underlined.

PROTEAN is commonly used for analysis of potential antigenic regions in the primary sequence of individual protein based on mainly the hydrophilicity of the query sequence [11]. Figure 5 shows the comparison between the performance of our RMA and PROTEAN. It was found that the epitopes of αRNase2, mAb 3C1 and D112-P123 Ab could be identified and predicted by both methods, and the experimental data further provided direct evidence of such correlation. In addition, the peptides "M_60_TCPSNKTR_68_" in RNase2 and "N_19_PPRC_23_" and "Y_98_ADRPG_103_" in RNase3 identified by RMA also appeared to have high potential to serve as specific and antigenic peptide antigens. The merit of RMA lies in its ability to provide additional information for rapid identification *in silico *of such antigenic peptides with high specificity.

### Performance analysis of RMA

**Figure 6:**
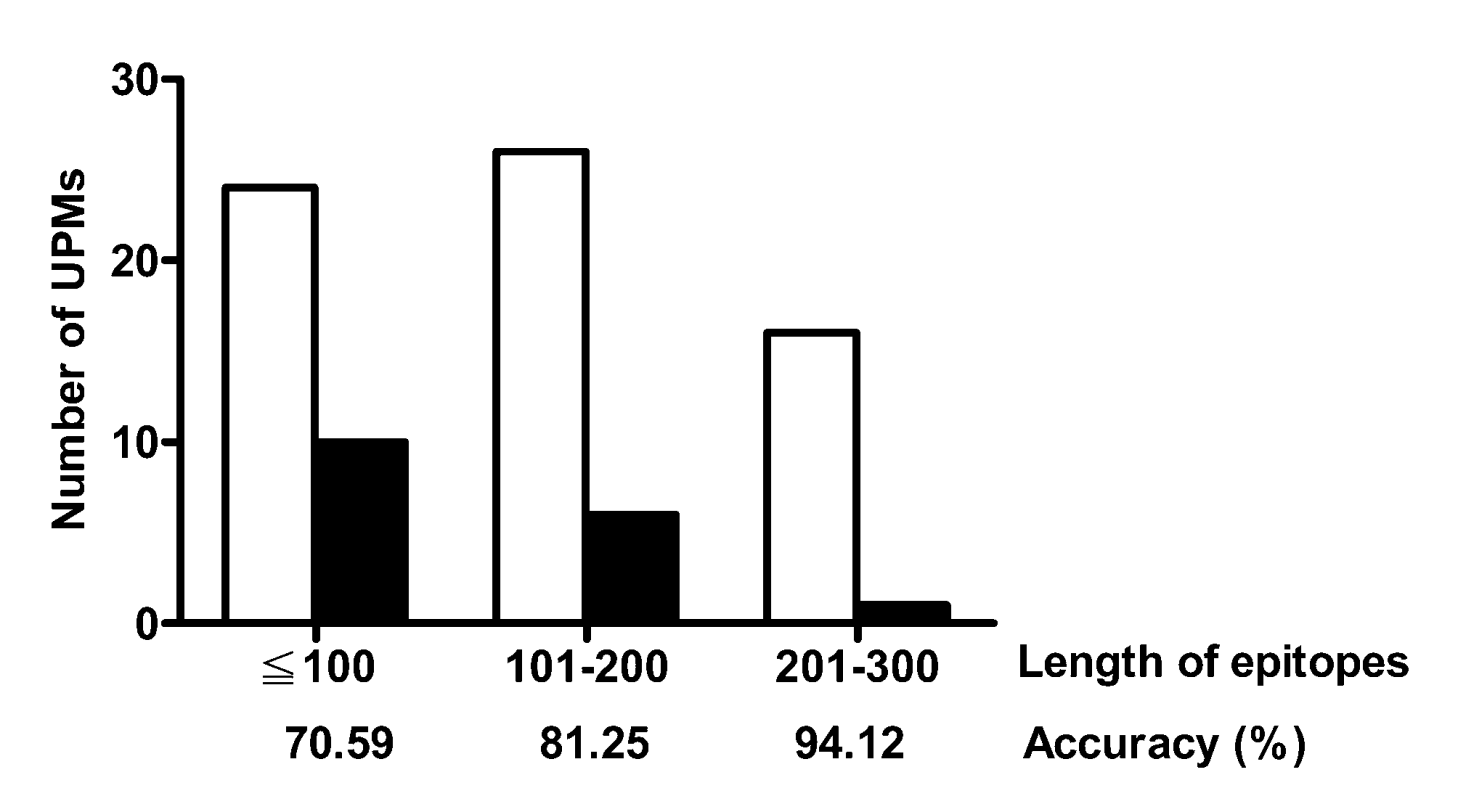
**Performance analysis of RMA**. The selected mAbs are divided into three groups based on the lengths of epitopes. The number of UPMs located and not located within the epitopes is expressed with open and solid column, respectively. The accuracy of correlating a UPM with an epitope is indicated below each group of mAbs.

Since no specialized database for the epitopes of mAbs against human proteins is currently available, the related information was retrieved from the website of Santa Cruz Biotechnology, Inc. which focused on the ongoing development of research antibodies [24]. Of 8398 items in the database, only 83 mAbs were derived from antigens classified into 63 human protein families containing 264 sequences. Based on the criteria described in the "Methods" section, each set of the family protein sequences was collected from GenBank and analyzed by RMA. It was found that 275 UPMs could be located within the recognition sites of 66 mAbs. Thus, the accuracy of matching at least one of the UPMs with the epitopes of the 83 selected mAbs in this case was calculated to be 79.52% (66/83). Figure 6 showed that as the lengths of the epitopes of the selected mAbs decreased from 300, 200, to 100 amino acid residues, the accuracy of a UPM being correlated with an epitope decreased from 94.12%, 81.25%, to 70.59%, respectively. Although the accuracy was length-dependent, an accuracy of higher than 70% could still be achieved.

## Discussion

We have demonstrated that RMA can identify UPMs in a number of protein families during multiple sequence comparison. Twenty-six sequences in four human protein families have been analyzed as shown in Table 3. In comparison with the 3-D structures of RNase1, 2, 3, 4 and 5, about 72% of the UPMs in the human RNaseA superfamily (18/25) was located in the loop regions of the structural surfaces. As for the biological functions of the UPMs, P64-F76 in RNase5 has been previously reported to be involved in the protein-cell interaction [13] and angiogenesis [25]. It should be noticed that in eight members of human RNaseA superfamily, only RNase5 possesses such angiogenetic activity, indicating that the uniqueness of P64-F76 correlates well with a specific function. In addition, the loop areas are known to possess the most flexible conformation and the residue compositions in loops are usually quite variable. Many peptides in the loop area have been reported to involve in molecular recognition by serving as the protein-protein interaction domains [26], enzyme-substrate binding sites [27], hormone binding motifs [28], or DNA binding motifs [29]. In our case, RMA successfully picked up the most characteristic features of these peptides among several protein families. Since the flexible conformation and variable residue composition in a loop region may provide additional specificity for recognition of Abs, identification of UPMs may further aid in epitope mapping as well as peptide antigen design.

In this study, we reported the analysis of three large protein families including EGFR, MMP, and Smad with respectively 39%-52%, 46%-78%, and 20%-92% pairwise sequence identity to test the practical application of RMA. The UPMs in the extracelluar domains of four members in EGFR family were subsequently analyzed. Unlike RNases, the extracellular domains in ErbB, ErbB2 and ErbB3 showed quite different conformations. In this particular data set, we found that 71% of the UPMs in EGFR family were located in the loops (Table 1). The UPM S350-K356 located within domain III (also named as L2) has been identified as an EGF binding domain on the extracellular domain of ErbB, the only member of the EGFR family that EGF recognizes (Figure 2, labeled in green) [14]. Combined with the structural features of human RNase and EGFR families, the UPMs provided additional information of many loops in the proteins with resolved structures. Thus, for the proteins without 3-D structural information, it is speculated that the UPMs may also be located in the loops to a considerable extent. Nevertheless, the possibility of a UPM being located in the well-defined α-helical or β-sheet structures cannot be excluded. As for the MMP superfamily, more than twenty members are currently classified, and several subfamilies are identified according to their domain compositions. It was thus necessary to classify the members of such a big protein family to several subgroups prior to RMA analysis. Accordingly, we have correlated two identified UPMs in MMP3 with the loop structures.

The experimental epitope mapping results suggest that some of the UPMs can serve as potential candidates to generate specific Abs to distinguish the highly conservative sequences in a protein family. In the past, sequential antigenic regions were determined based on the protrusion index, accessibility, mobility, charged amino acids or hydrophilic regions at the protein surface [9,30-32]. However, a good antigenic region may not specifically distinguish one protein from the other members in the same protein family. Our RMA has been demonstrated to efficiently discriminate the accumulation of unique features of a peptide fragment to distinguish different regions in a protein family. Although eight members of human RNaseA superfamily share high similarity, identification by RMA has given thirty UPMs of four to sixteen amino acids in length, among which at least three were demonstrated to serve as epitopes for αRNase2, mAb 3C1 and D112-P123 Ab (Table 1). In our case, theoretically 975 UPMs of eight residues in length in the human RNaseA superfamily can be identified. Therefore, RMA techniques have at least increased the probability of finding a UPM in RNaseA superfamily from 1/975 to 1/30. Likewise, the probability of finding such UPMs in human EGFR, MMP, and Smad families can be raised from 1/5087, 1/2784, and 1/3707 up to 1/39, 1/31, and 1/24, respectively. In ErbB2, a UPM H42-Q51 was located within the epitope of αErbB2 *N*-term (P36-Q51) at the first α-helix in domain I (Table 1) [33]. This Ab can recognize the structural surface of the well-folded extracellular domain. In addition, both αMMP1 and αMMP3 can probe denatured and well-folded proteins. Although 3-D structures containing the epitopes for αMMP1 and αMMP3 are not resolved yet, it is speculated that these regions are also exposed at the surfaces.

As for cross species protein analysis, the identity between bpRNaseA and hRNase1 was 68.8%. In these two highly conservative homologues, six identified UPMs of each RNase were localized in the corresponding positions in their primary sequences. Structurally, 83.3% of these regions were included in the loop areas of the resolved 3-D structures of bpRNaseA and hRNase1. Interestingly, except the first UPM, the loop coverage values of the second to the sixth UPMs in bpRNaseA are nearly the same as those of hRNase1 (Table 2), indicating that the loop areas of these two proteins are the most distinguishable. Similarly, the analysis of MsbAs by RMA revealed that the majority of the identified UPMs were located in the loop areas. Hence, RMA has been demonstrated to be useful to analyze homologous proteins from different species in addition to different proteins in one family in a particular species. The analysis of homologous proteins among different species may reveal the residue variation during molecular evolution. To our knowledge, it occurs sometimes that an antibody was not successfully produced as expected by injecting the immunized animals with a designed peptide or protein, probably due to lack of the uniqueness in the antigenic sequence [34]. Our RMA can identify the UPMs that do not exist in the homologous protein in the immunized animals; hence it can be used to provide an initial screening for potential epitope design to raise the probability of obtaining polyclonal antibodies against synthetic peptides.

In comparison with other motif-finding methods, MEME (Multiple Em for Motif Elicitation) system can discover the motifs with highly conserved regions in a group of related protein sequences. It represents motifs as position-dependent letter-probability matrices which describe the probability of each possible letter at each position in the pattern [35,36]. MAST (Motif Alignment & Search Tool) can search the biological sequence databases for sequences that contain one or more known motifs which are represented as position-dependent scoring matrices that describe the score of each possible letter at each position in the pattern [37]. The MASIA algorithm recognizes the common patterns and properties in multiple aligned protein sequences. It converts a sequence to a properties matrix that can be scanned in both vertical and horizontal steps. Consistent patterns are recognized based on the statistical significance of their occurrence [38]. PROSITE is a document database which collected a large number of biologically meaningful motifs that described as patterns or profile [39]. The main characteristics of these four sequence prediction algorithms are determination of the motifs with similar features. We have specifically analyzed the eight human RNase A sequences using PROSITE. It was found that the biological motifs such as potential phosphorylation sites and glycosylation sites identified by PROSITE were actually located within the conservative regions, none of them was identified as a UPM by RMA analysis. We have also matched all the biological motifs or pattern matrices in the PROSITE database with the proteins in PDB database. It was found that 35.31%, 24.68%, 0.64% and 39.36% of the PROSITE patterns were located at the α-helix, β-sheet, turn, and undetermined regions, respectively. Unlike the RMA results, only a few PROSITE patterns were located at the loops. Our RMA reinforces the non-conserved patterns and extracts the motifs with more than 8 residues. These UPMs are demonstrated to be correlated well with the loops exposed on the structural surface, or regions that are known to be able to serve as molecular recognition sites. It is conceivable that such unique sequence-dependent functional characteristics may be detected by our method, although the novel functional properties still remain to be explored. Evolutionally, the random unique sequence pieces are expected to be evenly distributed or randomly scattered in the entire protein sequences by statistical estimation. They are not specifically located in the loops. If a particular random mutation occurs in a position that is crucial for maintaining the structure integrity or catalytic function of a protein family, either the preservation of the mutation during evolution is disfavoured, or a dysfunctional protein would be generated to cause disease. On the contrary, the UPMs identified by our method are evolutionarily preserved sequence motifs, hence apart from the conserved regions; the UPMs tend to be located in loops and may be correlated with different functions of members in a protein family.

RMA is proved to be useful for finding characteristic sequence motifs in highly conservative members of one protein family. The UPMs may have the potential to be used as minivaccines for protecting animals from pathogenic infections. In general, the peptide-induced anti-peptide Ab must specifically recognize the desired infectious pathogen, but not cross-react to others. The UPMs identified by RMA (Figure 5, labeled in blue) can be directly compared with the antigenic regions predicted by PROTEAN (Figure 5, labeled as red star), such that the most specific and antigenic peptide antigen may be allocated (Table 1 and Table 2, labeled as #). In a large-scale analysis on mAbs with known epitopes on members of human protein families, RMA demonstrated higher than 70% accuracy in terms of correlating UPMs with epitopes of mAbs (Figure 6). Recently, Ecale Zhou *et al*. developed computational approaches employing a combination of structure- and sequence-based alignments for identification of the antibody/protein binding pockets on the surface of ricin A chain [40]. Structure alignment software and modified pocket-finding algorithm were required for extracting the residue-residue correspondence as well as for the determination of the conserved or unique binding pocket in the target molecules. Unlike the aforementioned method, our RMA directly identified and merged the non-gapped primary unique patterns; neither MSA nor structural information was prerequisite prior to the operation.

## Conclusion

Taken together, we have developed a bioinformatic tool to facilitate identification of UPMs located mostly in the loops or on the surface in members of a protein family. The structural features of some peptides are correlated with molecular recognition including antigen-antibody, protein-protein, protein-ligand and protein-cell interactions. Our method provides an effective and efficient evaluation of sequence, structural and functional studies that allow biologists to examine their protein sequences of interest prior to experiments such as peptide antigen design and mutagenesis.

## Methods

### Development of a Reinforced Merging Algorithm

**Figure 7:**
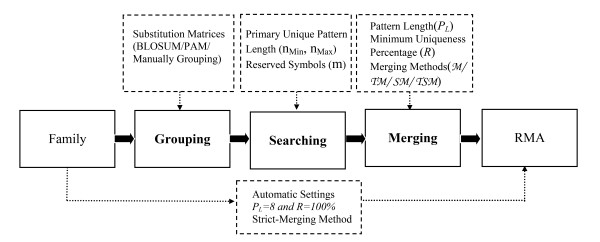
**System configuration and relative parameter settings**. Three main modules labeled in bold constitute the RMA system, and the dotted box above each of the main module describes the selected functional parameters.

The aim of this study is to construct the deterministic UPMs from a group of sequences among protein superfamilies by merging short primary unique patterns. Non-gapped primary unique patterns are obtained from a set of sequences and defined as possessing at least one symbol different with respect to all possible segments in other sequences. Bottom-up merging methodologies were applied to analyze the primary unique patterns and construct the uniqueness features from local and neighboring characteristics. There are three main modules designed in the RMA system including Grouping, Searching, and Merging modules. The system configuration and parameter settings in each module are depicted in Figure 7.

#### (A) Grouping module

The main purpose of the grouping module is to discriminate the tolerant features from UPM representation. Because the possible substitutions in different amino acids from observed frequencies in algorithms on related proteins are well-defined [41,42], we provide a fundamental clustering algorithm that assembles 20 amino acids into several independent groups. Users are able to assign threshold parameters and referred scoring matrices. A substitution matrix can be selected from standard BLOSUM/PAM matrices or new ones can be created based on their own aligned block database. In this module, the traditional hierarchical methods are employed to cluster groups based on the substitution matrix and threshold values. Amino acids grouped in the same set are treated as possessing similar characteristics and substituted by a common symbol. For examples, if we select the BLOSUM62 matrix and set the threshold parameter as 2, the module provides the grouping results ((A), (R, K), (N), (D, Q, E), (C), (G), (H, F, W, Y), (I, L, M, V), (P), (S), (T)), where amino acids within the same cluster represent matching states of identity. In addition, users are allowed to specify the number of groups and the contents of grouped amino acids based on different considerations.

#### (B) Searching module

In this module, we adopted the Boyer Moore matching algorithm [43] to extract primary unique patterns to reduce the time complexity. Primary unique patterns are defined as the basic elements for merging operations. Because each set of grouped amino acids are represented by a unique symbol, the algorithm examines all candidate patterns and extracts the positional information of primary unique patterns by scanning all substituted sequences. If the clustering results are less than 20 groups from the previous module, the tolerant feature of uniqueness will be discriminated and fewer primary unique patterns can be extracted from the family set. In addition to adopting clustered results to describe the features of uniqueness, this module also provides a statistical analysis to show the level of determined characteristics for each extracted unique pattern. Users are able to assign the mismatch number, which represents the tolerant criteria for primary unique patterns and should be less than the length of a primary unique pattern. If there are *N *sequences in a family set *Z*, and *Z*_*I *_is represented as the *I*^*th *^sequence in *Z*. The length of a primary unique pattern is *n *and reserved *m *symbols for each pattern, i.e. allow (*n-m*) mismatches from matching processes, then the representative level of uniqueness can be defined as the following equation,

R(Pnm[ZI,j])=1−1C(n,m)∑k=1C(n,m)〈Pn,km⌊ZI,j⌋〉−1N−1     (1)
 MathType@MTEF@5@5@+=feaafiart1ev1aaatCvAUfKttLearuWrP9MDH5MBPbIqV92AaeXatLxBI9gBaebbnrfifHhDYfgasaacH8akY=wiFfYdH8Gipec8Eeeu0xXdbba9frFj0=OqFfea0dXdd9vqai=hGuQ8kuc9pgc9s8qqaq=dirpe0xb9q8qiLsFr0=vr0=vr0dc8meaabaqaciaacaGaaeqabaqabeGadaaakeaacqWGsbGudaqadaqaaiabdcfaqnaaDaaaleaacqWGUbGBaeaacqWGTbqBaaGcdaWadaqaaiabdQfaAnaaBaaaleaacqWGjbqsaeqaaOGaeiilaWIaemOAaOgacaGLBbGaayzxaaaacaGLOaGaayzkaaGaeyypa0JaeGymaeJaeyOeI0YaaSaaaeaacqaIXaqmaeaacqWGdbWqcqGGOaakcqWGUbGBcqGGSaalcqWGTbqBcqGGPaqkaaWaaabCaeaadaWcaaqaamaaamaabaGaemiuaa1aa0baaSqaaiabd6gaUjabcYcaSiabdUgaRbqaaiabd2gaTbaakmaagmaabaGaemOwaO1aaSbaaSqaaiabdMeajbqabaGccqGGSaalcqWGQbGAaiaawcp+caGL7JpaaiaawMYicaGLQmcacqGHsislcqaIXaqmaeaacqWGobGtcqGHsislcqaIXaqmaaaaleaacqWGRbWAcqGH9aqpcqaIXaqmaeaacqWGdbWqcqGGOaakcqWGUbGBcqGGSaalcqWGTbqBcqGGPaqka0GaeyyeIuoakiaaxMaacaWLjaWaaeWaaeaacqaIXaqmaiaawIcacaGLPaaaaaa@6B63@

where we define the set of all primary unique descriptors with pattern length *n *in *Z*_*I *_by *P*_*n *_[*Z*_*I*_,·]. Its *j*^th ^primary unique descriptor is denoted as *P*_*n *_[*Z*_*I*_, *j*]. Pn,km
 MathType@MTEF@5@5@+=feaafiart1ev1aaatCvAUfKttLearuWrP9MDH5MBPbIqV92AaeXatLxBI9gBaebbnrfifHhDYfgasaacH8akY=wiFfYdH8Gipec8Eeeu0xXdbba9frFj0=OqFfea0dXdd9vqai=hGuQ8kuc9pgc9s8qqaq=dirpe0xb9q8qiLsFr0=vr0=vr0dc8meaabaqaciaacaGaaeqabaqabeGadaaakeaacqWGqbaudaqhaaWcbaGaemOBa4MaeiilaWIaem4AaSgabaGaemyBa0gaaaaa@3309@[*Z*_*I*_, *j*] represents a set of collection from the *j*^th ^unique pattern, which allows (*n-m*) mismatched symbols for matching processes, and Pnm
 MathType@MTEF@5@5@+=feaafiart1ev1aaatCvAUfKttLearuWrP9MDH5MBPbIqV92AaeXatLxBI9gBaebbnrfifHhDYfgasaacH8akY=wiFfYdH8Gipec8Eeeu0xXdbba9frFj0=OqFfea0dXdd9vqai=hGuQ8kuc9pgc9s8qqaq=dirpe0xb9q8qiLsFr0=vr0=vr0dc8meaabaqaciaacaGaaeqabaqabeGadaaakeaacqWGqbaudaqhaaWcbaGaemOBa4gabaGaemyBa0gaaaaa@30CA@[*Z*_*I*_, *j*] is its *k*^th ^tolerant pattern. The *C*(*n*, *m*) is the total number of possible combinatorial patterns set for reserved *m *symbols from a unique pattern with length *n*, and 〈Pnm
 MathType@MTEF@5@5@+=feaafiart1ev1aaatCvAUfKttLearuWrP9MDH5MBPbIqV92AaeXatLxBI9gBaebbnrfifHhDYfgasaacH8akY=wiFfYdH8Gipec8Eeeu0xXdbba9frFj0=OqFfea0dXdd9vqai=hGuQ8kuc9pgc9s8qqaq=dirpe0xb9q8qiLsFr0=vr0=vr0dc8meaabaqaciaacaGaaeqabaqabeGadaaakeaacqWGqbaudaqhaaWcbaGaemOBa4gabaGaemyBa0gaaaaa@30CA@[*Z*_*I*_, *j*]〉 ∊ [1, *N*] is the number of appearance of the *k*^th ^tolerant unique pattern shown in family set *Z*. It is trivial that the value of 〈Pnm
 MathType@MTEF@5@5@+=feaafiart1ev1aaatCvAUfKttLearuWrP9MDH5MBPbIqV92AaeXatLxBI9gBaebbnrfifHhDYfgasaacH8akY=wiFfYdH8Gipec8Eeeu0xXdbba9frFj0=OqFfea0dXdd9vqai=hGuQ8kuc9pgc9s8qqaq=dirpe0xb9q8qiLsFr0=vr0=vr0dc8meaabaqaciaacaGaaeqabaqabeGadaaakeaacqWGqbaudaqhaaWcbaGaemOBa4gabaGaemyBa0gaaaaa@30CA@[*Z*_*I*_, *j*]〉 is greater than or equal to 1, since at least one sequence from set *Z *containing the primary unique pattern *P*_*n *_[*Z*_*I*_, *j*] must exist. If all the *k *tolerant unique patterns Pnm
 MathType@MTEF@5@5@+=feaafiart1ev1aaatCvAUfKttLearuWrP9MDH5MBPbIqV92AaeXatLxBI9gBaebbnrfifHhDYfgasaacH8akY=wiFfYdH8Gipec8Eeeu0xXdbba9frFj0=OqFfea0dXdd9vqai=hGuQ8kuc9pgc9s8qqaq=dirpe0xb9q8qiLsFr0=vr0=vr0dc8meaabaqaciaacaGaaeqabaqabeGadaaakeaacqWGqbaudaqhaaWcbaGaemOBa4gabaGaemyBa0gaaaaa@30CA@[*Z*_*I*_, *j*] can be matched in each sequence in *Z*, then 〈Pnm
 MathType@MTEF@5@5@+=feaafiart1ev1aaatCvAUfKttLearuWrP9MDH5MBPbIqV92AaeXatLxBI9gBaebbnrfifHhDYfgasaacH8akY=wiFfYdH8Gipec8Eeeu0xXdbba9frFj0=OqFfea0dXdd9vqai=hGuQ8kuc9pgc9s8qqaq=dirpe0xb9q8qiLsFr0=vr0=vr0dc8meaabaqaciaacaGaaeqabaqabeGadaaakeaacqWGqbaudaqhaaWcbaGaemOBa4gabaGaemyBa0gaaaaa@30CA@[*Z*_*I*_, *j*]〉 = *N *for all k, and it follows that *R*(Pn,km
 MathType@MTEF@5@5@+=feaafiart1ev1aaatCvAUfKttLearuWrP9MDH5MBPbIqV92AaeXatLxBI9gBaebbnrfifHhDYfgasaacH8akY=wiFfYdH8Gipec8Eeeu0xXdbba9frFj0=OqFfea0dXdd9vqai=hGuQ8kuc9pgc9s8qqaq=dirpe0xb9q8qiLsFr0=vr0=vr0dc8meaabaqaciaacaGaaeqabaqabeGadaaakeaacqWGqbaudaqhaaWcbaGaemOBa4MaeiilaWIaem4AaSgabaGaemyBa0gaaaaa@3309@[*Z*_*I*_, *j*]) = 0%. This describes a special condition in which each possible tolerant pattern of the primary unique descriptor Pn,km
 MathType@MTEF@5@5@+=feaafiart1ev1aaatCvAUfKttLearuWrP9MDH5MBPbIqV92AaeXatLxBI9gBaebbnrfifHhDYfgasaacH8akY=wiFfYdH8Gipec8Eeeu0xXdbba9frFj0=OqFfea0dXdd9vqai=hGuQ8kuc9pgc9s8qqaq=dirpe0xb9q8qiLsFr0=vr0=vr0dc8meaabaqaciaacaGaaeqabaqabeGadaaakeaacqWGqbaudaqhaaWcbaGaemOBa4MaeiilaWIaem4AaSgabaGaemyBa0gaaaaa@3309@[*Z*_*I*_, *j*] can be discovered in all other sequences of *Z*. Therefore, its representative percentage of primary uniqueness will be decreased to 0%. On the other hand, if all of the combinatorial tolerant patterns from Pn,km
 MathType@MTEF@5@5@+=feaafiart1ev1aaatCvAUfKttLearuWrP9MDH5MBPbIqV92AaeXatLxBI9gBaebbnrfifHhDYfgasaacH8akY=wiFfYdH8Gipec8Eeeu0xXdbba9frFj0=OqFfea0dXdd9vqai=hGuQ8kuc9pgc9s8qqaq=dirpe0xb9q8qiLsFr0=vr0=vr0dc8meaabaqaciaacaGaaeqabaqabeGadaaakeaacqWGqbaudaqhaaWcbaGaemOBa4MaeiilaWIaem4AaSgabaGaemyBa0gaaaaa@3309@[*Z*_*i*_, *j*] cannot be found from other family sequences, then 〈Pnm
 MathType@MTEF@5@5@+=feaafiart1ev1aaatCvAUfKttLearuWrP9MDH5MBPbIqV92AaeXatLxBI9gBaebbnrfifHhDYfgasaacH8akY=wiFfYdH8Gipec8Eeeu0xXdbba9frFj0=OqFfea0dXdd9vqai=hGuQ8kuc9pgc9s8qqaq=dirpe0xb9q8qiLsFr0=vr0=vr0dc8meaabaqaciaacaGaaeqabaqabeGadaaakeaacqWGqbaudaqhaaWcbaGaemOBa4gabaGaemyBa0gaaaaa@30CA@[*Z*_*I*_, *j*]〉 = 1 for *k *∊ [1, *C*(*n*, *m*)] and it follows that by *R*(Pn,km
 MathType@MTEF@5@5@+=feaafiart1ev1aaatCvAUfKttLearuWrP9MDH5MBPbIqV92AaeXatLxBI9gBaebbnrfifHhDYfgasaacH8akY=wiFfYdH8Gipec8Eeeu0xXdbba9frFj0=OqFfea0dXdd9vqai=hGuQ8kuc9pgc9s8qqaq=dirpe0xb9q8qiLsFr0=vr0=vr0dc8meaabaqaciaacaGaaeqabaqabeGadaaakeaacqWGqbaudaqhaaWcbaGaemOBa4MaeiilaWIaem4AaSgabaGaemyBa0gaaaaa@3309@[*Z*_*I*_, *j*]) = 100%. This describes another extreme case in which the program provides all of the possible tolerant patterns for approximate matching, and the primary unique pattern of Pn,km
 MathType@MTEF@5@5@+=feaafiart1ev1aaatCvAUfKttLearuWrP9MDH5MBPbIqV92AaeXatLxBI9gBaebbnrfifHhDYfgasaacH8akY=wiFfYdH8Gipec8Eeeu0xXdbba9frFj0=OqFfea0dXdd9vqai=hGuQ8kuc9pgc9s8qqaq=dirpe0xb9q8qiLsFr0=vr0=vr0dc8meaabaqaciaacaGaaeqabaqabeGadaaakeaacqWGqbaudaqhaaWcbaGaemOBa4MaeiilaWIaem4AaSgabaGaemyBa0gaaaaa@3309@[*Z*_*I*_, *j*] still possesses 100% unique representative percentage. These calculated quantitative percentages represent the level of uniqueness and range from 0% to 100%. All of the primary unique patterns with different representative percentages will be sent to the next module to perform merge operations based on their neighbouring conditions and threshold settings for the representative levels of uniqueness.

#### (C) Merging module

The merging operation is proposed to enhance the discrepancies in a family set and emphasize the neighboring relationships instead of the traditional concatenation operation. Two matched primary unique patterns in sequence *Z*_*I *_can be merged if they possess overlapping symbols in *Z*_*I *_and both are satisfied the criteria of the minimum representative percentage. Given any two segments *u*_*1*_and *u*_*2 *_with length *n*_*1 *_and *n*_*2*_, the merged result is denoted by *w *and constructed by

w(i)={u1(i)if 1≤i≤n1;u2(i−n1+l)if n1+1≤i≤n1+n2−l.     (2)
 MathType@MTEF@5@5@+=feaafiart1ev1aaatCvAUfKttLearuWrP9MDH5MBPbIqV92AaeXatLxBI9gBaebbnrfifHhDYfgasaacH8akY=wiFfYdH8Gipec8Eeeu0xXdbba9frFj0=OqFfea0dXdd9vqai=hGuQ8kuc9pgc9s8qqaq=dirpe0xb9q8qiLsFr0=vr0=vr0dc8meaabaqaciaacaGaaeqabaqabeGadaaakeaacqWG3bWDcqGGOaakcqWGPbqAcqGGPaqkcqGH9aqpdaGabaqaauaabaqaciaaaeaacqWG1bqDdaWgaaWcbaGaeGymaedabeaakiabcIcaOiabdMgaPjabcMcaPaqaaiabdMgaPjabdAgaMjaaykW7cqaIXaqmcqGHKjYOcqWGPbqAcqGHKjYOcqWGUbGBdaWgaaWcbaGaeGymaedabeaakiabcUda7aqaaiabdwha1naaBaaaleaacqaIYaGmaeqaaOGaeiikaGIaemyAaKMaeyOeI0IaemOBa42aaSbaaSqaaiabigdaXaqabaGccqGHRaWkcqWGSbaBcqGGPaqkaeaacqWGPbqAcqWGMbGzcaaMc8UaemOBa42aaSbaaSqaaiabigdaXaqabaGccqGHRaWkcqaIXaqmcqGHKjYOcqWGPbqAcqGHKjYOcqWGUbGBdaWgaaWcbaGaeGymaedabeaakiabgUcaRiabd6gaUnaaBaaaleaacqaIYaGmaeqaaOGaeyOeI0IaemiBaWMaeiOla4caaaGaay5EaaGaaCzcaiaaxMaadaqadaqaaiabikdaYaGaayjkaiaawMcaaaaa@6C29@

where *l *is the number of overlapping symbols between *u*_*1 *_and *u*_*2*_, and *l *is strictly greater than 0. If *u*_*1 *_and *u*_*2 *_do not contain any overlapping symbols between them, then the merging operation will not be performed, and both will retain their original relationship. It is obvious that if *u*_*1 *_and *u*_*2 *_are merged with respect to *Z*_*I*_, the length of the merged segment *w *is strictly less than the concatenated string *uv*. To reinforce the strength of uniqueness of the merged patterns, a strict merging operation is also provided to make sure each subsegment of the merged pattern still possess unique properties. Assume two primary unique patterns with same length *n *are merged. The strict merging requirements are satisfied only when the lengths of the overlapping symbols are equal to the primary pattern length minus one or the length of the merged segment is equal to *n+1*. Therefore, strictly merged unique patterns are constructed using fundamental unique descriptors and reserve the most unique characteristics in sequences. After obtaining all candidate merged segments, the system provides a trimming function which returns a substring from each merged segment with the symbols stripped off the beginning and the end. This function is achieved by evaluating the beginning and ending (*n-1*) symbols respectively from merged unique pattern set. The (*n-1*) symbols will be trimmed off when they are matched with another merged unique. Through these three designed modules, UPMs of each sequence from a protein family set will be allocated efficiently. After reinforced processing, these merged UPMs satisfy the tolerance and representative level of uniqueness criteria. The RMA is available free of charge for academic use [44,45] and a supplementary document for guidelines and examples of the RMA system is also provided [46].

### Analyses of protein sequences by RMA and localization of the identified UPMs

The protein sequences could be selected according to the following criteria: (1) If the protein of interest belongs to a protein family classified by the sequence similarity, each member of this protein family is selected from GenBank and saved as FASTA format. (2) If the protein of interest does not belong to any protein family, its sequence should be compared with other protein sequences in the database by BLASTp software or others and all the sequences with more than 20% identity should be selected and saved as FASTA format.

In this paper, the protein sequences of human RNaseA, EGFR, MMP, and Smad families (Table 3) were saved in FASTA format and entered into the RMA for analysis. The length of the lower bound, upper bound and primary unique pattern were all set as 3. After the searching and merging processes, the merged UPMs with length greater than 8 were identified. The unique regions, trimmed residues, and conserved regions were labeled in blue, orange, and black, respectively. The positions of all UPMs identified by RMA were localized and colour-coded in the resolved 3-D structures of each query sequence employing WebLab ViewerPro 4.0 (Molecular Simulations Inc.). For cross species analysis, RNaseAs from *Bos Taurus *(*B. Taurus*) and *Homo sapiens *(*H. sapiens*), and MsbAs, one of the largest superfamilies of proteins characterized by a highly conserved adenosine triphosphate (ATP) binding cassette (ABC), from *Escherichia coli *(*E. coli*), *Vibro cholera *(*V. cholera*), *Gloeobacter violaceus *(*G. violaceus*), *Shewanella oneidensis *(*S. oneidensis*), and *Bradyrhizobium japonicum *(*B. japonicum*) (Table 4) were analyzed by the same processes.

### Protein expression and generation of mAb

The recombinant clones were transformed into *E. coli *BL21 (DE3) and expressed in the presence of 0.5 mM IPTG. After homogenization, the RNase3 was purified by His-Bind^® ^metal chelating chromatography under denaturing conditions according to the manufacturer's protocol (Novagen). The generation of mAb was carried out as described [47]. Hybridomas producing anti-RNase3 mAb were obtained by indirect enzyme-linked immunosorbent assay screening with purified recombinant RNase3 as the antigen. The mAb was purified from the hybridoma supernatant using the Montage^® ^Antibody Purification Kit (Millipore).

### Western blotting

After electrophoresis, the proteins were transferred onto a polyvinylidene fluoride membrane (Pall). After blocking, hybridizing with the first Ab, washing, and probing with the secondary Ab, the target proteins were visualized using the SuperSignal West Pico (Pierce) or 3,3'-Diaminobezidine tetrahydrochloride (Amersco).

### Analysis of protein sequences by PROTEAN

Each protein sequence was saved in PROTEIN format using EditSeq in DNAStar and executed by PROTEAN [11]. The sequential sequences were selected as the antigenic index higher than 0.5 and compared with the UPMs identified by RMA. Between potential antigenic regions and UPMs, the coverage greater than 70% was labeled in Table 1 and 2.

### Large-scale RMA analysis

To analyze the performance of RMA in large-scale, the input sequences were selected by the following steps. First, all information related to mAbs in website of Santa Cruz Biotechnology, Inc. was retrieved [24] and the mAbs containing descriptions about the exact recognition sites (epitopes) were selected. Then the corresponding antigen sequences belonging to human protein families were collected from GenBank and saved as FASTA format. Each family protein sequence set with pairwise identity higher than 20% was individually analyzed by RMA with default parameters as previously described.

## Abbreviations

GFP, green fluorescent protein; EGFR, epidermal growth factor receptor; mAb, monoclonal antibody; MBP, maltose binding protein, MMP, matrix metalloproteinase; MSA, multiple sequence alignment; RMA, reinforced merging algorithms; RNA, ribonucleic acid; RNase, ribonuclease; Smad, Sma-and-Mad related protein; TGF-β, transforming growth factor-β; UPM, unique peptide motif

## Authors' contributions

HTC carried out biological experiments, participated in the sequence analysis and drafted the whole manuscript. TWP participated in the design of the RMA program and its coordination. TF performed the statistic analysis. BHS participated in the design and modification of RMA program. PCW participated in the initial design of RMA program. CYT helped to modify the informatics' part of this manuscript. CTC helped to analyze the PROSITE pattern. SHL participated in the molecular cloning. MDTC conceived this study and helped to draft the manuscript. All authors read and approved the final manuscript.

## Figures and Tables

**Table 1:** Characterization of UPMs in protein families identified by RMA

protein family^a^	UPM	Position	loop coverage (%)^b^	Molecular recognition^c^	Refs
**RNaseA**^d^					
Rnase1	MDSDSSPSSSSTY^#^	13–25	70		[48]
	GQGNCYKSNSSMHITD^#^	68–83	31		[48]
	KERHI^#^	102–106	0		[48]
	FDASVEDST^#^	120–128	50		[48]
Rnase2	QQCTNAMQVI	21–30	60		[49]
	MTCPSNKTR^#^	60–68	100		[49]
	HSGSQ^#^	73–77	50	αRNase2^e^	[49],^f^
	TPANMFYI^#^	101–108	0		[49]
Rnase3	ISLNPPRCTIAMR^#^	16–28	100		[50]
	RCPHNRTLNN^#^	61–70	90	mAb 3C1^e^	[50],^f^
	RSRFRVP^#^	73–79	29		[50]
	INPGAQ	86–91	83		[50]
	YADRPGRRF^#^	98–106	0		[50]
	PRDSPRY^#^	116–122	100	D112-P123 Ab^e^	[12, 50]
Rnase4	VHPEETGG^#^	13–20	64		[51]
	RKMTLYH	32–38	86		[51]
	IHEDIWNIR	46–54	0		[51]
	CSTTNIQ^#^	57–63	100		[51]
	EGVVK	73–77	0		[51]
	RDTGS^#^	82–86	50		[51]
	IASTRRVVI^#^	97–105	0		[51]
Rnase5	THFLT	7–11	0		[25]
	KAICEN^#^	54–59	100		[25]
	PHRENLRISKSSF^#^	64–76	14	protein-cell interaction^g^	[13, 25]
	WPPCQY^#^	89–94	60		[25]
**EGFR**					
ErbB	CQGTSN^#^	31–36	50		[14]
	LSNYDA	144–149	0		[14]
	SINATNI	350–356	100	EGF binding	[14]
	RTDLHAF	414–420	100		[14]
	TKQHGQF^#^	430–436	100		[14]
	TSGQKT^#^	483–488	33		[14]
ErbB2	HLDMLRHLYQ	42–51	0	αErbB2 *N*-term^e^	[33] Stratagene Cat: B50175
	ILWKDIFHK	144–152	100		[33]
	GLGMEHL	321–327	100		[33]
	APLQPEQ	367–373	100		[33]
	HNGAYSL	414–420	71		[33]
	CFVHTVPWDQLFR	452–464	100		[33]
ErbB3	YIEKNDKLC^#^	129–137	33		[52]
	FAHEAECFSCH^#^	524–534	0		[52]
**MMP**^**d**^					
MMP1	RGDHRD^#^	66–71	-^g^		
	SHSTDIG^#^	128–134	-		
	GRSQNPVQPI^#^	162–171	-		
	WAVQGQN	250–256	-		
	RTVKHI	273–278	-	αMMP1^*e*^	Santa Cruz Cat: sc-21731
	YKRSM^#^	304–308	-		
	MIAHD	315–319	-		
	HGTRQYKF	341–348	-		
	KTKRILTLQKAN^#^	351–362	-		
MMP3	QWTKDTTGT^#^	103–111	100		[15]
	TDLTRFR	145–151	100		[15]
	TEPVP	178–182	-		
	GTPANC^#^	186–191	-		
	KSLRKLEP^#^	218–225	-	αMMP3^d^	Santa Cruz Cat: sc-21732
	PKQIAE^#^	324–329	-		
**Smad**					
Smad4	MDNMSITNT	1–9	-		
	TTNGAH	62–67	-	DNA binding	[20]
	KHVKYCQY	110–117	-		
	IQTIQH	179–184	-		
	HPGHYWPVH	297–305	-		
	HKIYPSAYIK	427–436	0		[18]
	AISLSA	481–486	0		[18]
	GPDYPRQSI^#^	510–518	100	Ski binding	[18]
	EVLHTMPIADPQPL	538–551	64	TGFβreceptor binding	[18]

^a ^The members of each protein family were analyzed by RMA. The proteins possessing resolved 3-D structures or epitope information are listed in column 1.

^b ^The loop coverage of each identified UPM was calculated and shown as the percentage localized within a loop in accordance with the 3-D structure.

^c ^The UPMs with known protein-molecule interaction are indicated.

^d ^The protein sequences do not include the signal peptides of RNases and pre-pro regions of MMPs.

^e^The epitopes of the antibodies containing or within the identified UPMs are indicated.

^f^The epitopes of the mAbs identified in this article are indicated.

^g^This UPM is responsible for the protein-cell interaction for the angiogenesis of RNase5.

^h^"-", No 3-D structural information is currently available.

^#^UPMs overlap with the potential antigenic regions by 70% as predicted by PROTEAN.

**Table 2:** Characterization of UPMs in homologues from different species identified by RMA

protein family^a^	UPM	position	loop coverage (%)^b^	Molecular recognition	Refs
**RNaseA**					
Bovine	TAAAKFE^#^	3–9	14		[53]
	KSRNLTKD^#^	31–38	63	αbpRNaseA^c^	[21, 53]
	AVCSQKNVA	56–64	56		[53]
	QSYSTMS	74–80	29		[53]
	ETGSSK^#^	86–91	67		[53]
	KTTQANK	98–104	0		[53]
Human	SRAKKFQ^#^	3–9	100, -^d^		[48]
	RRRNMTQG^#^	31–38	75		[48]
	NVCFQEKVT	56–64	56		[48]
	KSNSSMH^#^	74–80	29		[48]
	LTNGSR^#^	86–91	67		[48]
	RTSPKER^#^	98–104	0		[48]
**MsbA**					
*Escherichia coli*	MHNDKD^#^	1–6	-^e^		[22]
	PSVMDS	273–278	100		[22]
	DVEFRN	341–346	67, -^d^		[22]
	RNINLKI	360–366	-		[22]
	HRGVY	568–572	-		[22]
*Vibrio cholera*	ADTYMIS	40–46	0		[23]
	ESNFL	60–64	100		[23]
	NHFMHM	106–111	83		[23]
	ADPVIQ	251–256	100		[23]
	RAELT	276–280	100		[23]
	GKYEAER^#^	331–337	100		[23]
	VDVKD^#^	342–346	20		[23]
	YQGKEK^#^	351–356	0		[23]

^a^The members of each protein family were analyzed by RMA. The proteins possessing resolved 3-D structures or epitope information are listed in column 1.

^b ^The loop coverage of each identified UPM was calculated and shown as percentage localized within a loop in accordance with the 3-D structure.

^c ^The epitope of the antibody containing or within the identified UPMs is indicated.

^d^The loop coverage is calculated based on the sequences located in the solved 3-D structures.

^e^"-", No 3-D structural information is currently available.

^#^UPMs overlap with the potential antigenic regions by 70% as predicted by PROTEAN.

**Table 3:** The sequences of human protein families for RMA analysis

Name	Accession No.	Name	Accession No.
RNaseA			
RNase1	NP_937878	RNase5	NP_001136
RNase2	AAG31583	RNase6	NP_005606
RNase3	NP_002926	RNase7	NP_115961
RNase4	P34096	RNase8	NP_612204
EGFR			
ErbB	NP_958441	ErbB3	AAH02706
ErbB2	AAA75493	ErbB4	A47253
MMP			
MMP1	NP_002412	MMP10	NP_002416
MMP3	NP_002413	MMP12	NP_002417
MMP8	NP_002415	MMP13	NP_002418
Smad			
Smad1	Q15797	Smad5	Q99717
Smad2	Q15796	Smad6	O43541
Smad3	P84022	Smad7	O15105
Smad4	Q13485	Smad9	O15198

**Table 4:** The sequences of protein families from different species for RMA analysis

Name	Species	Accession No.
Pancreatic RNaseA	*Homo sapiens*	NP_937878
	*Bos Taurus*	P61823
MsbA	*Escherichia coli*	1JSQA
	*Vibro cholera*	1PF4D
	*Gloeobacter violaceus*	NP_927081
	*Shewanella oneidensis*	NP_718380
	*Bradyrhizobium japonicum*	NP_772572
